# Impact of Alzheimer’s Disease on Oral Health and Regional Variations in Long-Term Care (2020-2024)

**DOI:** 10.1093/geroni/igaf122.3029

**Published:** 2025-12-31

**Authors:** Joohyun Chung, Weidong Wang, Jing Qian

**Affiliations:** University of Massachusetts Amherst, Amherst, Massachusetts, United States; University of Massachusetts Amherst, Amherst, Massachusetts, United States; University of Massachusetts Amherst, Amherst, Massachusetts, United States

## Abstract

Around 1.3 million older adults in long-term care (LTC) facilities are at high risk for poor oral health, often neglected, particularly among those with Alzheimer’s Disease and Related Dementias (ADRD). This study examines the prevalence of oral health issues, such as tooth loss and gum disease, in LTC residents at various stages of ADRD. It also explores the impact of Alzheimer’s on oral health and regional disparities using state-level data from the Minimum Data Set (MDS) from 2020 to 2024. Using a non-experimental, quantitative, longitudinal study design, the research analyzes time trends in the prevalence of oral health problems across various ADRD stages. Descriptive statistics and mixed logistic models were used to examine the relationship between ADRD stages (intact/borderline impairment, moderate, and severe) and oral health outcomes. The study also explores regional differences in the prevalence of tooth loss and gum disease across U.S. The findings show that ADRD significantly affects oral health. Residents with intact/borderline ADRD exhibit poorer oral health, while moderate and severe ADRD significantly increases the prevalence of tooth loss/ gum disease (p < 0.001). States like Texas, California, and Florida have seen declines in tooth loss rates, while Kentucky and West Virginia continue to experience high rates. For gum disease, reductions were noted in California, Georgia, and Florida, while Hawaii and Montana saw sharp increases. These results highlight the need for targeted oral health interventions, especially in states with high prevalence rates, to improve care for ADRD residents and address regional disparities in oral health outcomes.

